# Thermoplastic Elastomer‐Reinforced Hydrogels with Excellent Mechanical Properties, Swelling Resistance, and Biocompatibility

**DOI:** 10.1002/advs.202414339

**Published:** 2025-02-07

**Authors:** Zhanqi Liu, Hechuan Zhang, Ruigang Zhou, Haiyang Gao, Yongchuan Wu, Yuqing Wang, Haidi Wu, Cheng Guan, Ling Wang, Longcheng Tang, Pingan Song, Huaiguo Xue, Jiefeng Gao

**Affiliations:** ^1^ School of Chemistry and Chemical Engineering Yangzhou University No 180, Road Siwangting Yangzhou Jiangsu 225002 China; ^2^ College of Veterinary Medicine Yangzhou University Yangzhou 225009 China; ^3^ Department of Chemistry College of Liberal Arts and Sciences University of Florida Gainesville FL 32611 USA; ^4^ School of Chemistry and Chemical Engineering Anqing Normal University Anqing 246011 China; ^5^ Key Laboratory of Organosilicon Chemistry and Material Technology of Ministry of Education College of Material Chemistry and Chemical Engineering Hangzhou Normal University Hangzhou 311121 China; ^6^ Centre for Future Materials University of Southern Queensland Springfield QLD 4350 Australia

**Keywords:** hydrogels, mechanical properties, phase separation, swelling resistance

## Abstract

Strong and tough hydrogels are promising candidates for artificial soft tissues, yet significant challenges remain in developing biocompatible, anti‐swelling hydrogels that simultaneously exhibit high strength, fracture strain, toughness, and fatigue resistance. Herein, thermoplastic elastomer‐reinforced polyvinyl alcohol (PVA) hydrogels are prepared through a synergistic combination of phase separation, wet‐annealing, and quenching. This approach markedly enhances the crystallinity of the hydrogels and the interfacial interaction between PVA and thermoplastic polyurethane (TPU). This strategy results in the simultaneous improvement of the mechanical properties of the hydrogels, achieving a tensile strength of 11.19 ± 0.80 MPa, toughness of 62.67 ± 10.66 MJ m^−3^, fracture strain of 1030 ± 106%, and fatigue threshold of 1377.83 ± 62.78 J m^−2^. Furthermore, the composite hydrogels demonstrate excellent swelling resistance, biocompatibility, and cytocompatibility. This study presents a novel approach for fabricating strong, tough, stretchable, biocompatible, and fatigue‐ and swelling‐resistant hydrogels with promising applications in soft tissues, flexible electronics, and load‐bearing biomaterials.

## Graphical abstract

Thermoplastic polyurethane (TPU)‐reinforced polyvinyl alcohol (PVA) hydrogels with excellent swelling resistance and biocompatibility are prepared through a combination of phase separation, wet‐annealing, and quenching. The enhanced crystallinity of PVA macromolecular chains and the interfacial interactions between PVA and TPU contribute to the simultaneous improvement of the mechanical properties including the tensile strength, toughness, and fatigue threshold of the hydrogels.

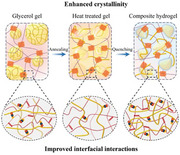

## 1 Introduction

Hydrogels have garnered significant interest from both industry and academia because of their intriguing properties, such as flexibility, tissue‐like mechanical characteristics, environmental friendliness, and their resemblance to the extracellular matrix. These features make hydrogels highly promising for tissue engineering applications.^[^ ^1^, ^2^, ^3^, ^4^, ^5^ ^]^ However, their weak mechanical properties and poor swelling resistance have severely limited their use, particularly in load‐bearing biomaterials.^[^ ^6^ ^]^

The composite strategy is widely employed to enhance the mechanical properties of hydrogels, often involving the introduction of additional components, such as polymers, ions, or nanofillers.^[^ ^7^, ^8^, ^9^, ^10^ ^]^ For instance, double networks are constructed to introduce energy dissipation for hydrogel toughening. Typically, the double network comprises contrasting structures. During stretching, the fracture of brittle network would dissipate a large amount of energy, while the stretchable network provides large deformation and deconcentrates the stress.^[^ ^11^ ^]^ Nevertheless, the strengthening and toughening are usually effective in a single cycle of mechanical loading, and the fatigue resistance of the hydrogels is low during cyclic loadings. In addition, the double network is usually constructed by sequential polymerization and chemical crosslinking, and the unreacted monomers, residual initiators, and crosslinkers may be cytotoxic, severely limiting their bio‐applications. Bonakdar and coworkers used prepolymer (polyester polyurethane) to chemically crosslink polyvinyl alcohol (PVA) in dimethyl sulfoxide (DMSO), and obtained composite organogels with the double network showed enhanced tensile strength, which was attributed to the rigidity of PU and elevated strength of the crosslinking bond. However, the PVA organogels possessed relatively small fracture strain, and the water take up percentage was still as high as 100%, despite the improved swelling resistance.^[^ ^12^ ^]^ Hofmeister effect is a well‐established method to tune the interaction between macromolecules and solvent, and salting out can promote the aggregation of macromolecules and thus facilitate gelation. It was reported that the ions for salting out generally improve the crystallinity of the PVA hydrogels while decreasing the adjacent distance between the crystalline domains, and the densified polymer network usually leads to enhancement of both strength and toughness.^[^ ^7^, ^13^ ^]^ Despite the strengthening and toughening effect, the ions inside the hydrogels easily diffuse outside in an aqueous media. Consequently, the hydrogels are swollen to a great degree and the mechanical properties are accordingly declined, making them difficult in practical applications, especially in an aqueous environment. Another universal reinforcement technique is based on the incorporation of nanofillers such as nanoparticles and nanofibers into the hydrogels.^[^ ^14^, ^15^, ^16^, ^17^, ^18^, ^19^, ^20^ ^]^ For instance, aramid nanofibers and cellulose nanofibers are often used to reinforce the hydrogels through interfacial hydrogen bonding, because the stress can effectively transfer from the soft macromolecular chains to the rigid nanofibers. However, the incorporation of nanofillers even at a low concentration would greatly increase the solution viscosity,^[^ ^21^ ^]^ complicating the processing of hydrogels. In addition, nanofillers are readily aggregated, and the large nanofiller agglomerates usually aggravate the mechanical properties of the hydrogels. Particularly, the elongation at break may decline dramatically.^[^ ^22^ ^]^

Despite advancements in composite hydrogels with enhanced mechanical properties, substantial challenges remain in developing mechanically strong yet tough, biocompatible bio‐nontoxic, and anti‐swelling hydrogels. Achieving concurrent enhancements in tensile strength, toughness, stretchability, and fatigue resistance remains particularly challenging. In this study, thermoplastic elastomer (thermoplastic polyurethane, TPU)‐reinforced hydrogels are prepared by the synergy of phase separation, wet‐annealing, and quenching. The increased crystallinity of the hydrogels and enhanced interfacial interactions between the phase‐separated TPU and PVA contribute to the overall improvement of mechanical properties. The composite hydrogels exhibit high tensile strength and toughness (up to 11.19 ± 0.80 MPa and 62.67 ± 10.66 MJ m^−3^, respectively) with impressively large fracture strain of 1030 ± 106%, and the fatigue threshold can also reach as high as 1377.83 ± 62.78 J m^−2^, respectively, showing great crack propagation resistance. In addition, the composite hydrogels exhibit remarkable swelling resistance due to the synergy of the crystalline domains of PVA and the hydrophobic TPU phase. Furthermore, no signs of rejection reaction or inflammation are observed when the composite hydrogels are introduced in animals, demonstrating biocompatibility and cytocompatibility.

## 2 Results and Discussion

**Figure 1:**
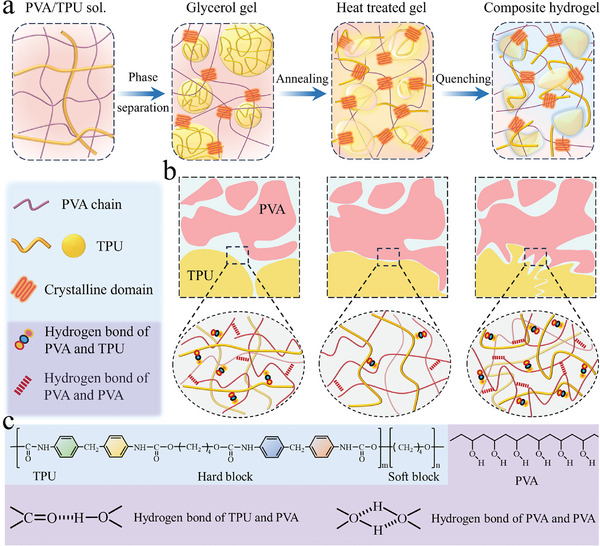
Schematic illustration of the preparation and microstructure of thermoplastic elastomer‐reinforced hydrogels. a) Fabrication process for the composite hydrogel; b) Evolution of interfacial morphology and interfacial interactions; c) Representation of the chemical structures involved in inter‐ and intra‐molecular interactions.

The preparation of thermoplastic elastomer‐reinforced hydrogels is schematically depicted in **Figure** **1**a. Here, a phase separation strategy was used to introduce thermoplastic polyurethane (TPU) into PVA hydrogels. PVA and TPU were initially dissolved in their good solvent, namely, dimethyl sulfoxide (DMSO) (Figure S1, Supporting Information). PU usually possesses hard and soft segments in its macromolecular chains, and interchain hydrogen bonds are formed between the hard segments or hard‐soft segments (Figure S2, Supporting Information). In the blend polymer solution, the interaction between the solvent and polymers dominates and exceeds the inter‐macromolecular interactions, leading to the disentanglement of both PVA and TPU macromolecular chains. Subsequently, the homogeneous blend solution is immersed in glycerol for solvent exchange. Note that glycerol is the poor solvent for PVA and non‐solvent for TPU, respectively. Consequently, gelation occurs. The gelation is caused by the PVA macromolecular chain aggregation and hence the formation of crystal domains as physical crosslinking points, because glycerol is a poor solvent for PVA, and the interaction between the macromolecular chains of PVA prevails during the solvent exchange. On the other hand, TPU precipitates as microspheres because of the non‐solvent induced phase separation. Finally, the phase separated TPU microspheres are uniformly distributed in the PVA/glycerol organogel. TPU with the microphase separation structure exhibits a unique combination of strength and stretchability.^[^ ^23^, ^24^ ^]^ Although the solvent exchange promotes the formation of the composite hydrogels, TPU is excluded from the PVA network, producing huge interfacial gaps while limiting interfacial areas. In addition, there are many amorphous regions with disordered PVA chains in the hydrogel, giving rise to a relatively low crystallinity of PVA macromolecules. Thus, wet‐annealing is used to tune the PVA conformation while at the same time improving the interfacial interactions between TPU and PVA. Annealing activates PVA macromolecular chain motions, leading to the enhancement of both crystallinity and chain entanglement.^[^ ^6^ ^]^ During annealing, the high boiling point of glycerol and robust PVA skeleton endow the composite organogel with sufficient mechanical strength and thermal stability, guaranteeing the structure's integrity. PVA macromolecule movement is activated and TPU is softened or even partially melted at such a high temperature, and the macromolecular chain rearrangement and conformation adjustment would disrupt instantaneously the inter/intra molecular hydrogen bonding. Finally, wet‐annealing densifies the polymeric network with improved inter‐chain hydrogen bonding between PVA and the crystallinity. During quenching, the temperature of the organogel decreases quickly while at the same time, glycerol is replaced by water, leading to the cooling induced contraction. As a result, macromolecular chain movement and the interfacial structure between PVA and TPU are frozen, and the improved hydrogen bonding, crystallinity, and interfacial adhesion are maintained in the finally obtained composite hydrogels. It is worth noting that multiple inter/intra macromolecular hydrogen bonding are present in the interface regions of the composite hydrogel (Figure 1b,c). The multiple hydrogen bonds and macromolecular entanglement endow the composite hydrogels with excellent mechanical properties.

To facilitate description, PVA/TPU/DMSO solution is denoted by L‐PVA_x_‐TPU_y_, where x and y represent the mass percentages of PVA and TPU in the solution, respectively. Hydrogels/organogels prepared by direct one step solvent exchange (either from DMSO to water or from DMSO to glycerol) were denoted by PVA_x_‐TPU_y_, Gly‐PVA_x_‐TPU_y_, respectively. All wet‐annealed gels were suffixed with the capital letter A, and hydrogels prepared by solvent exchange (DMSO to glycerol), wet annealing, and quenching were denoted by PVA_x_‐TPU_y_‐AQ or PVA_x_‐AQ.

The rheological behavior of different hydrogels is shown in Figures S3 and S4 (Supporting Information). The storage modulus (G′) in the linear region at a fixed oscillation strain of 0.1% was consistently an order of magnitude higher than the loss modulus (G″) for all hydrogels, indicating solid‐like and elastic‐dominated properties (Figure S3, Supporting Information).^[^ ^25^, ^26^ ^]^ The higher the PVA or TPU concentration, the larger both G′ and G″ of the composite hydrogels. For example, G′ and G″ of PVA_15_‐TPU_8_‐AQ were comparable to those of PVA_23_‐AQ but higher than those of PVA_15_‐TPU_5_‐AQ within the frequency range of 0.1–100 rad s^−1^ (Figure S4a, Supporting Information). However, with the increase of the oscillation strain, PVA_23_‐AQ showed a quicker decrease in both G′ and G″ than PVA_15_‐TPU_8_‐AQ (Figure S4b, Supporting Information).

**Figure 2:**
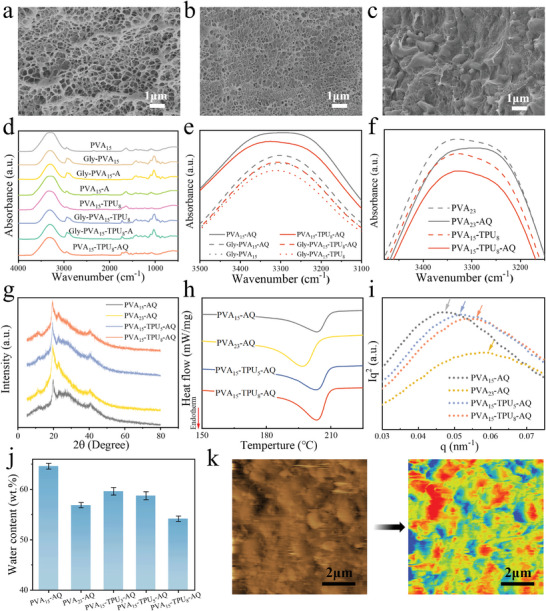
Morphology and microstructure of the hydrogels. SEM images of a) PVA_15_‐AQ, b) PVA_23_‐AQ, and c) PVA_15_‐TPU_8_‐AQ. d) ATR‐FTIR spectra and e, f) enlarged spectra of the stretching vibrations of O‐H. g) XRD patterns. h) DSC curves. i) SAXS curves. j) Water content. k) AFM image and phase image of PVA_15_‐TPU_8_‐AQ.

The elastomer TPU can effectively regulate the conformation of PVA macromolecules, densify the PVA backbone and improve the hydrogel's mechanical properties. As the PVA content increased, the gel network of PVA_23_‐AQ became denser compared to that of PVA_15_‐AQ (**Figure** **2**a,b). PVA_15_‐TPU_8_ as the controlled sample prepared by one‐step solvent exchange (DMSO to water) method possessed similarly porous structure with PVA_15_‐AQ (Figure S5, Supporting Information), and TPU microspheres were exposed and loosely attached onto the skeleton of PVA_15_‐TPU_8_ with limited contact areas, indicating the weak interface interactions and hence inferior mechanical properties. Interestingly, the pores disappeared inside both PVA_15_‐TPU_5_‐AQ and PVA_15_‐TPU_8_‐AQ (Figure 2c and Figure S6, Supporting Information), and the densified PVA network where TPU microspheres were uniformly embedded, was formed. Also, wet‐annealing promoted the fusion of many TPU microspheres. In addition, TPU were tightly wrapped by the PVA matrix, exhibiting the enhanced interface area and adhesion. It was also worth noting that the microspheres became larger and irregular with the increase of TPU content because more phase separated TPU microspheres tended to coalesce during the wet‐annealing. There are abundant hydroxyl groups on PVA macromolecular chains while plenty of amine groups on TPU macromolecular chains, which can form the inter molecular hydrogen bonding and hence enhance the interfacial interactions.

Fourier transform infrared (FTIR) was used to characterize the hydrogen bonding of the composite hydrogels (Figure 2d). A broad peak at around 3300 cm^−1^, which corresponds to the stretching vibration of the hydroxyl group or the amine groups, are present in all the hydrogels, and this characteristic peak is often used to analyze the hydrogen bonding of hydrogels.^[^ ^27^, ^28^, ^29^ ^]^ It can be found that the peaks of composite hydrogels were shifted to higher wavenumbers than those of the single‐component PVA gels, indicating the formation of possible hydrogel bonding between PVA and TPU (Figure 2e). Also, wet‐annealing induced the shift of the characteristic peak to the low wavenumbers, resulting from the enhanced inter and intra macromolecular interactions (Figure 2f). As shown in Figure S7 (Supporting Information), the C─O absorption peak at 1143 cm^−1^ is related to the formation of crystalline domains in PVA.^[^ ^6^ ^]^ It was observed that the introduction of TPU intensified the C‐O absorption peak. In addition, the C‐O absorption peaks of PVA_23_‐AQ and PVA_15_‐TPU_8_‐AQ were found to be stronger than those of PVA_23_ and PVA_15_‐TPU_8_, indicating that the wet annealing can promote PVA crystallization. The above results demonstrated the advantages of wet annealing‐quenching for optimizing interfacial compatibility and hydrogen bonding.

X‐ray diffraction (XRD) was used to study the crystallization behavior of the hydrogels (Figure 2g). The diffraction peaks at 2θ = 19.7° of the composite hydrogels were stronger than those of PVA_15_‐AQ but weaker than those of PVA_23_‐AQ.^[^ ^30^, ^31^ ^]^ This indicated that phase separation promoted the crystallization of PVA to a certain extent, while the higher PVA concentration was beneficial to the macromolecular crystallization. The crystal size (D) was calculated by Scherrer's formula (see the experimental section), and D of PVA_15_‐AQ was only 6.8 nm, whereas the crystal sizes of PVA_23_‐AQ and PVA_15_‐TPU_8_‐AQ were increased to 8.8 and 8.0 nm, respectively (Figure S8, Supporting Information). This result indicates that TPU phase separation promoted the growth of PVA crystals to a certain degree.

DSC tests were conducted to quantitatively calculate the crystallinity of these hydrogels. As shown in Figure 2h, there were evident endothermic peaks for the hydrogels, indicating the crystalline regions were damaged during heating induced melting.^[^ ^32^, ^33^ ^]^ According to their melting enthalpies, the crystallinities of PVA_15_‐AQ, PVA_15_‐TPU_5_‐AQ, PVA_15_‐TPU_8_‐AQ, and PVA_23_‐AQ in the dry states were 21.9%, 24.5%, 27.8%, and 30.3%, respectively, and 7.7%, 10.0%, 12.8%, and 13.0%, respectively, in the swollen state (Figure S9, Supporting Information). The phase separation of TPU from the solution extrudes the surrounding PVA chains, which is favorable for crystallization. In addition, a high PVA concentration facilitates the formation of PVA‐rich phases and hence the crystalline regions. SAXS tests were also performed to examine the distance between the adjacent crystalline regions (Figure 2i). The long period was decreased from 13.4 nm (PVA_15_‐AQ) to 11.3 nm (PVA_15_‐TPU_8_‐AQ) with the increase of TPU content, which was consistent with those results from the XRD and DSC tests. Thus, the results indicate that TPU phase separation has a positive impact on enhancing the crystallinity of PVA and facilitating the formation of compact crystal domains.

Water content is a crucial factor that affects the mechanical properties of hydrogels.^[^ ^34^, ^35^ ^]^ As exhibited in Figure 2j, PVA_15_‐AQ exhibited the highest water content of 65%, while the water content of PVA_23_‐AQ decreased to 57% because of the increased PVA concentration and thus denser 3D polymeric skeleton inside the hydrogel. PVA_15_‐TPU_8_‐AQ, despite its lower crystallinity than PVA_23_‐AQ, showed the lowest water content of 54% due to the introduction of a hydrophobic TPU phase in the hydrogel. The phase separation can also be verified by the atomic force microscopy (AFM) images of PVA_15_‐TPU_8_‐AQ (Figure 2k), two phases were observed, and many TPU microdomains were coalesced and separated from the continuous PVA matrix., which was consistent with the observation from SEM images. The blue region corresponds to the continuous PVA hydrogel network with a lower modulus, while the red region represents the discontinuous TPU phases with a higher modulus, and the high modulus region was contributed by granular TPU. As shown in Figure S10 (Supporting Information), PVA_15_‐AQ showed a flatter and smoother surface with a lower modulus compared with that of PVA_15_‐TPU_8_‐AQ.

**Figure 3:**
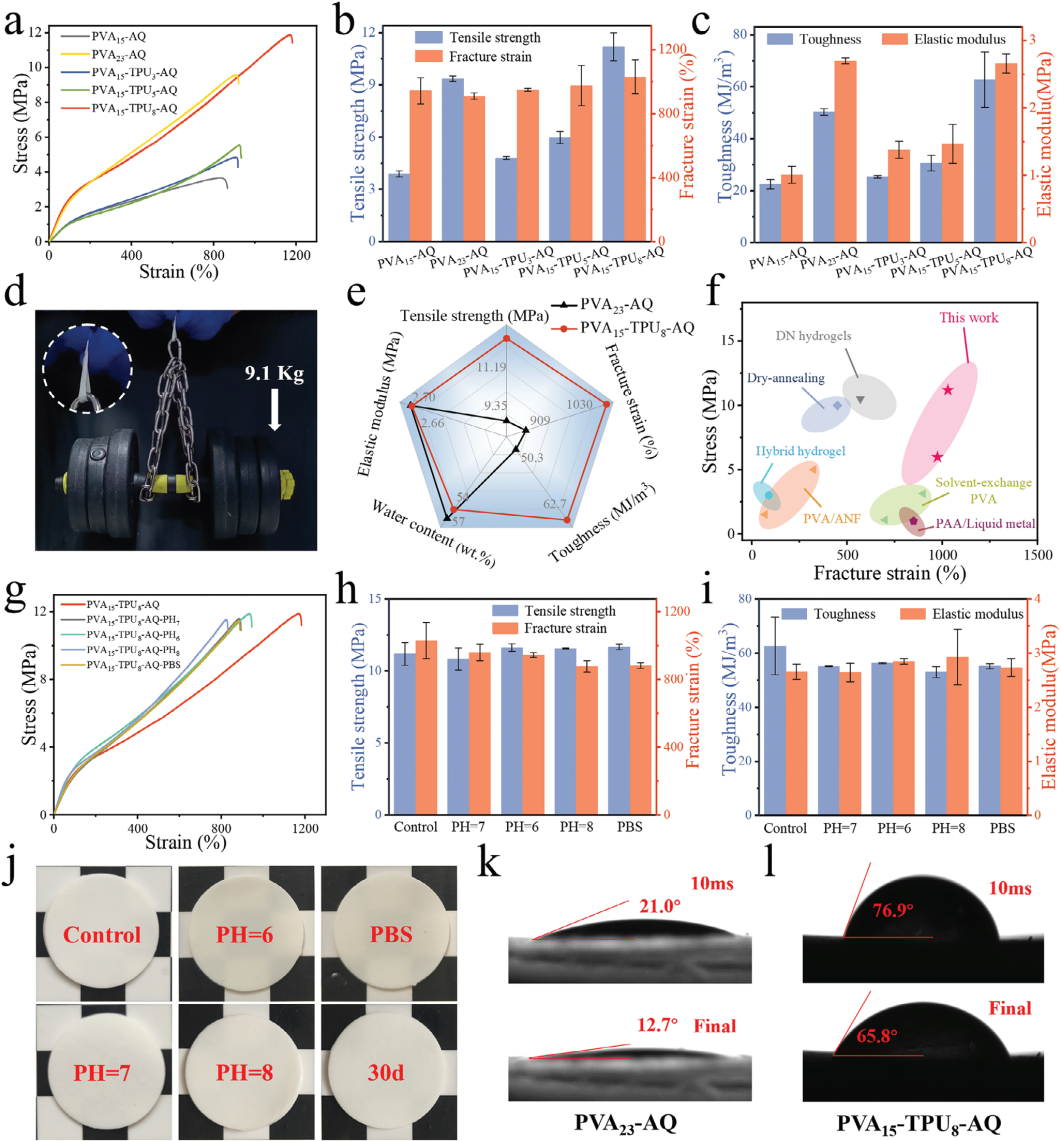
Mechanical properties and swelling resistance of the hydrogels. a) Uniaxial stretching stress‐strain curves. Summarized b) tensile strength and fracture strain and c) toughness and elastic modulus of the hydrogels. d) Images of PVA_15_‐TPU_8_‐AQ withstanding the 9.1 kg dumbbell. e) Comparison of the mechanical properties of PVA_15_‐TPU_8_‐AQ and PVA_23_‐AQ. f) Comparative plot of tensile strength versus fracture strain for PVA_15_‐TPU_5_‐AQ and PVA_15_‐TPU_8_‐AQ alongside other reported hydrogels. g) Stress‐strain curves and h, i) histograms summarizing the mechanical properties of PVA_15_‐TPU_8_‐AQ before and after swelling in different aqueous media for 30 days. j) Photographs of PVA_15_‐TPU_8_‐AQ before and after swelling in different aqueous media for 30 days. k) Water contact angle of PVA_23_‐AQ and l) PVA_15_‐TPU_8_‐AQ.

The composite hydrogels exhibited excellent mechanical performance. The typical stress‐strain curves and the summarized various mechanical properties are shown in **Figure** **3**a–c, TPU enhanced the overall mechanical properties of hydrogels. The higher the TPU content, the better the mechanical properties. Particularly, PVA_15_‐TPU_8_‐AQ exhibited overwhelming advantages in mechanical performance over other hydrogels. The tensile strength, fracture strain, elastic modulus and toughness of PVA_15_‐AQ were 3.89 ± 0.16 MPa, 943 ± 82%, 1.00 ± 0.12 MPa and 22.51 ± 1.79 MJ m^−3^, respectively, and were improved to 5.97 ± 0.34 MPa, 975 ± 127%, 1.47 ± 0.29 MPa and 30.61 ± 3.07 MJ m^−3^ for PVA_15_‐TPU_5_‐ AQ, and further greatly enhanced to 11.19 ± 0.80 MPa, 1030 ± 106%, 2.66 ± 0.14 MPa, and 62.67 ± 10.66 MJ m^−3^ for PVA_15_‐TPU_8_‐ AQ. PVA_15_‐TPU_8_‐AQ could easily support a dumbbell with a total weight of 9.1 kg (Figure 2d). It was worth noting that wet‐annealing and quenching greatly enhanced the mechanical properties of both PVA_15_ and PVA_15_‐TPU_8_ (Figure S11, Supporting Information). Generally, the rigid nanofillers such polymer nanofibers, carbon nanomaterials, and inorganic nanofillers are incorporated to hydrogels to improve their mechanical properties. However, these nanofillers are easily aggregated in the hydrogels even at a low concentration, and it is also challenging to achieve strong interfacial interaction between the nanofillers and the hydrogel matrix. In many cases, the enhancement of strength and modulus is at the cost of the elongation at break and toughness.^[^ ^17^, ^36^ ^]^ In this study, TPU microspheres are phase separated from the solution and hence uniformly distributed in the organogel, but they are also isolated with the PVA skeleton, leading to very small interfacial contact areas. Fortunately, wet‐annealing of organogel with high thermal stability improves both the interfacial contact areas and interfacial adhesion. Also, the coalesce occurs between the softened or even partially melted TPU microspheres, which is beneficial to the stress de‐concentration and crack propagation resistance. For comparison, the TPU foam was obtained using the same method for the preparation of the composite hydrogel. As shown in Figure S12, (Supporting Information), the tensile strength of TPU_8_‐AQ was 0.48 ± 0.13 MPa, higher than that of TPU_8_ (0.23 ± 0.02 MPa). This result indicates that annealing could partially fuse the skeleton of the foam and cause the densification of TPU polymer network, giving rise to the enhancement of the mechanical properties. As shown in Figure 3e, PVA_15_‐TPU_8_‐AQ demonstrated comprehensive improvement in mechanical properties, by comparison with PVA_23_‐AQ prepared with the same solid concentration. The mechanical properties including the tensile strength and fracture strain of the TPU‐reinforced hydrogels in this work are in the leading position compared to those of other reported hydrogels such as DN hydrogels, PVA/ANF and PAA/liquid metal hydrogels (Figure 3f).^[^ ^10^, ^15^, ^29^, ^33^, ^37^, ^38^ ^]^ The increased crystallinity produces more physical crosslinking points that can withstand larger stress, and the rigid yet stretchable TPU reinforces PVA hydrogels through effective stress transfer via interfacial hydrogen bonding. It is worth noting that the stretchability of TPU as well as strong interfacial interactions allow large deformation of TPU with the hydrogel during stretching, and the macromolecular chain entanglement in amorphous regions enhances the stretchability (i.e., fracture strain) of the hydrogels. The slip between macromolecular chains as well as between the TPU and PVA interface and damage to the crystal regions consume a large amount of energy. Therefore, simultaneous enhancement in strength and toughness is achieved for the composite hydrogels.

In many circumstances, hydrogels are swollen in the aqueous environment because of the hydrophilicity of the macromolecular chains, leading to the structure instability and decline of mechanical properties.^[^ ^39^ ^]^ Therefore, swelling resistance is required to maintain the initial mechanical properties and reliability of hydrogels working in complex aqueous environments. To examine the anti‐swelling performance of the hydrogels, PVA_15_‐TPU_8_‐AQ was immersed in different solutions including deionized water, buffer solution, and solutions with different pH values for 30 days. A comprehensive evaluation of the tensile strength, elongation at break, toughness, and modulus showed that the composite hydrogel almost maintained its mechanical properties with slightly declined fracture strain and toughness (Figure 3g–i). In addition, PVA_15_‐TPU_8_‐AQ kept its original shape and appearance with little change in its volume and structure (Figure 3j). These results verify the anti‐swelling resistance of the composite hydrogels and can be used in a variety of aqueous media. Since the composite hydrogels were finally obtained from the solvent exchange (i.e., quenching in water), the mechanically strong hydrogels reached an almost equilibrium state in water, and cannot absorb water anymore. Furthermore, the hydrophobic TPU can retard water permeation into the composite hydrogel.^[^ ^12^ ^]^ As displayed in Figure 3k,l, the water droplet quickly diffused on the surface PVA_23_‐AQ, and the contact angle (CA) decreased from the initial 21.0°–12.7°. By contrast, CA was as high as 76.9° after the droplets were in contact with PVA_15_‐TPU_8_‐AQ for 10 ms, and finally maintained at 65.8°, much higher than that of PVA_23_‐AQ. In summary, the robust polymeric skeleton with numerous crystalline regions as the crosslinking points and the decreased hydrophilicity are responsible for the outstanding swelling resistance of the composite hydrogels.

**Figure 4:**
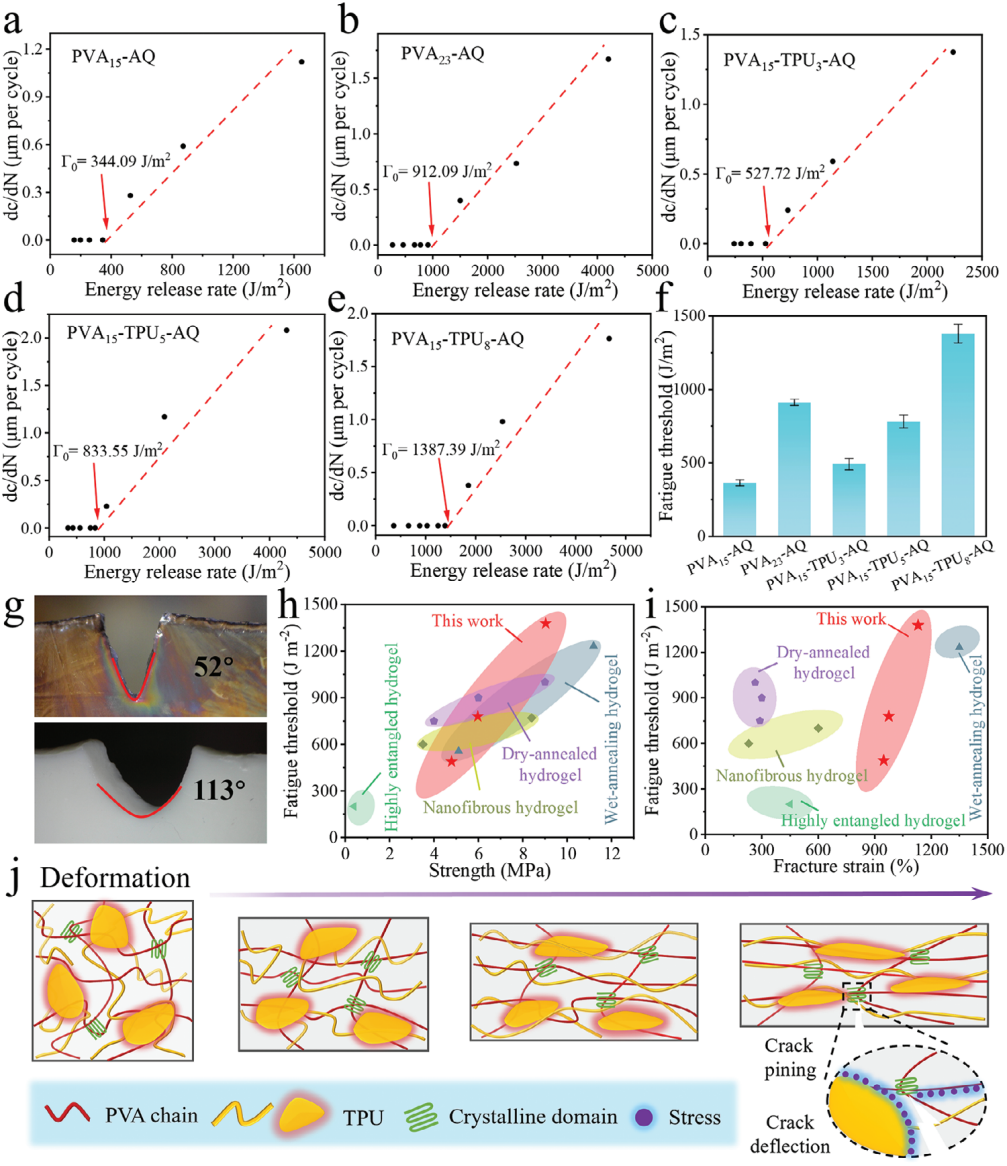
Crack propagation resistance of the composite hydrogels. Crack extension rate versus applied energy release rate for a) PVA_15_‐AQ, b) PVA_23_‐AQ, c) PVA_15_‐TPU_3_‐AQ, d) PVA_15_‐TPU_5_‐AQ, e) PVA_15_‐TPU_8_‐AQ, and f) summary of their fatigue thresholds. g) Images of the notches of PVA_15_‐AQ and PVA_15_‐TPU_8_‐AQ. h) Fatigue threshold versus tensile strength and i) fatigue threshold versus fracture strain of the composite hydrogels in this work compared to other tough hydrogels. j) Schematic representation of the fatigue resistance mechanism.

Generally, polymer material exhibits both viscosity and elasticity, and thus the mechanical relaxation cannot be ignored during their mechanical deformations and the effect of energy dissipation mechanisms on fatigue resistance should not be underestimated.^[^ ^18^, ^40^, ^41^ ^]^ Enhancement of the crystalline domains within the hydrogel can increase the energy required for the fatigue crack initiation and fracture per unit area, thereby significantly increasing its fatigue threshold.^[^ ^33^ ^]^ **Figure** **4**a–f exhibited the measured fatigue threshold (FT) for various hydrogels. It can be found that FT increases with the concentration of PVA or TPU, and PVA_15_‐TPU‐AQ possesses higher FT than PVA_15_‐AQ. For instance, FT was only 344.09 J m^−^^2^ for PVA_15_‐AQ, while it greatly increased to 912.09 J m^−2^ for PVA_23_‐AQ. Surprisingly, the fatigue threshold of PVA_15_‐TPU_8_‐AQ reached 1387.39 J m^−^^2^, exceeding that of most other reported isotropic hydrogels. The notch of PVA_15_‐AQ was gradually enlarged during stretching and the angle between the two sides of the notch was 52°. By comparison, the angle of the notch for PVA_15_‐TPU_8_‐AQ was extended to 113° (Figure 4g), indicating that TPU‐reinforced hydrogel can effectively blunt crack and dissipate energy.^[^ ^42^ ^]^ After 30 000 stretching‐releasing cycles, no crack extension or redirection for PVA_15_‐TPU_8_‐AQ was observed (Figure S13, Supporting Information), indicating great fatigue resistance. Figure S14 (Supporting Information) showed the stress versus time curve of notched PVA_15_‐TPU_8_‐AQ with a strain of 70%, and the maximum stress showed a gradual decline and then varied little during the long‐term stretching‐releasing test. The tensile stress‐strain curves overlap from the 6000th cycle. Compared with other tough isotropic hydrogels.^[^ ^6^, ^33^, ^41^, ^43^ ^]^ TPU‐reinforced PVA hydrogels showed almost the largest fatigue threshold while at the same time possessing high fracture strain and tensile strength (Figure 4h,i), demonstrating the unique advantage of the synergy of phase separation, wet‐annealing, and quenching for preparation of strong, tough and anti‐fatigue hydrogels.

As mentioned, nanofillers are often used to strengthen the hydrogels, and the strong interfacial interaction should be guaranteed, otherwise, cracks can easily propagate along the weak interface, leading to low energy dissipation and quick failure. In addition, large agglomerates from the nanofiller aggregation may become structure defects, thereby aggravating the mechanical properties, especially the stretchability of hydrogels. Fortunately, the phase separation enables the uniform distribution of TPU microspheres in the hydrogels, while wet‐annealing eliminates the interfacial gaps and improves the interfacial interactions. Also, the annealing and quenching promote the crystallization and entanglement of PVA macromolecules. The mechanism for the strengthening and fatigue resistance of the composite hydrogels is illustrated schematically in Figure 4j. During stretching, both PVA macromolecules and TPU tend to align along the stretching direction. Stress is transferred from one polymer chain to other chains and from weak amorphous regions to strong crystalline regions through slip between PVA macromolecules. In addition, the strong hydrogen bonding and interfacial adhesion enable the effective stress transfer from soft PVA gel to rigid yet stretchable TPU, and the interfacial slip and friction also dissipate huge energy. The crystalline domains represent strong and stiff crosslinking regions of the PVA hydrogels, and the higher crystallinity gives rise to a larger energy dissipation. On the other hand, the straightening of the highly entangled PVA macromolecular chains and alignment of TPU phases contributed to the large deformation capability and thus high fracture strain. The stretching promotes the PVA macromolecular chain assembly into strong fibrous bundles and the TPU phases to fibers. The 2D SAXS pattern showed that the regular diffraction ring gradually turned into a sharp diffraction ring when PVA_15_‐TPU_8_‐AQ was stretched, indicating the orientation of the macromolecular chains (Figure S15, Supporting Information). Also, the SEM image of PVA_15_‐TPU_8_‐AQ stretched by 100% confirmed that TPU embedded in the gel network forms coalesced fibers along the stretching direction (Figure S16, Supporting Information). Both the PVA fibrous bundles and TPU fibers can deconcentrate the stress and deflect the cracks. Furthermore, they are able to prevent crack propagation through the fiber bridging and pull‐out effect. In conclusion, the multi‐scale structure and multiple interfacial interactions give rise to the excellent mechanical properties of the composite hydrogels with simultaneous improved tensile strength, toughness, fracture strain, and fatigue threshold. This coordinated interplay of multiscale interactions is essential for achieving a high fatigue threshold.

**Figure 5:**
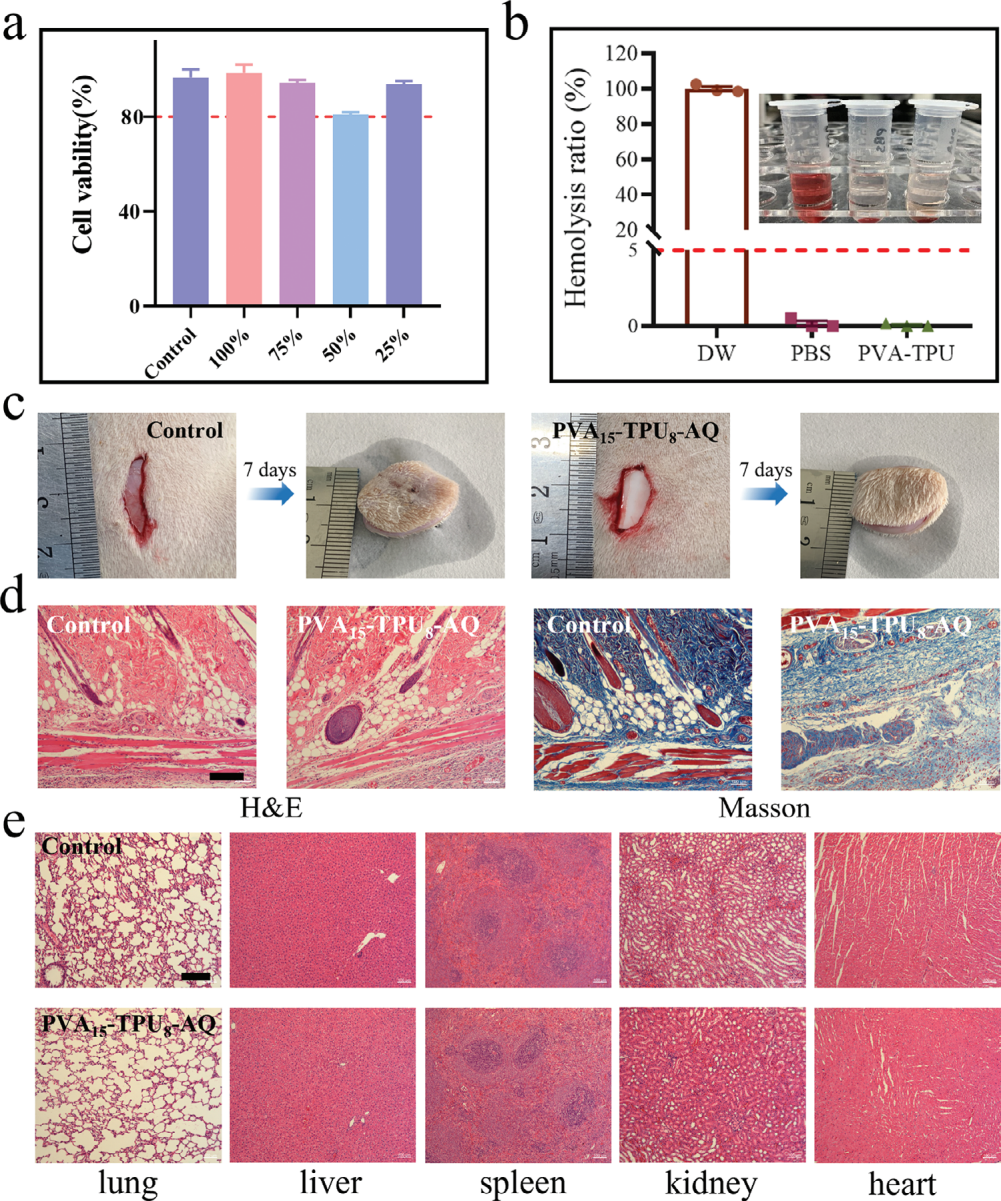
Biocompatibility of the composite hydrogel. a) Cytocompatibility evaluation of the composite hydrogel, demonstrating cell viability. b) Hemocompatibility analysis, showing minimal hemolysis and blood compatibility. c, d) Representative images of the control group and the composite hydrogel implanted subcutaneously on the backs of rats, along with H&E and Masson staining of skin tissue in the implantation area (scale bar: 200 µm). e) H&E staining of major organs from rats, indicating no significant tissue abnormalities or inflammatory responses (scale bar: 200 µm).

Conventional hydrogels exhibit relatively poor mechanical properties and poor swelling resistance, limiting their use in long‐term load‐bearing applications especially in the aqueous environment. TPU‐reinforced PVA hydrogels, on the other hand, offer promising opportunities in the field of tissue engineering due to their excellent mechanical properties, resistance to swelling and fatigue, and their capability to work in complex in vivo conditions. Biocompatibility is one of the most important factors evaluating the safety of hydrogels for biomedical use.^[^ ^44^ ^]^ First, cytocompatibility is an important index to evaluate the biocompatibility. The cytocompatibility of hydrogels was analyzed by CCK‐8 method using the hydrogel leachate on L929 cells (**Figure** **5**a).^[^ ^45^ ^]^ It was found that the cellular activity in the leachate at different concentrations from 25% to 100% was greater than 70%, and according to the national standard GB/T16886.5‐2017, it can be concluded that the composite hydrogel has good cytocompatibility. Besides, hydrogels may come into contact with damaged skin. From the biocompatibility point of view, hydrogels must have good hemocompatibility.^[^ ^46^ ^]^ In order to evaluate the blood compatibility of hydrogels, we performed the hemolysis test. The results in Figure 5b showed that the hemolysis rate of the composite hydrogel was less than 5%, indicating its good blood compatibility. These results indicate that the composite hydrogels can be further used for in vivo studies without safety concerns. Thus, we performed the staining analysis of skin tissues to evaluate the safety of the hydrogel in vivo (Figure 5c). H&E staining showed a small amount of inflammatory cell infiltration near the implantation site, which was similar to the results of the control group (Figure 5d, (Figure S17, Supporting Information). In addition, Masson staining showed relatively dense and abundant collagen deposition in the composite hydrogel group, also similar to results in the control group (Figure S18, Supporting Information). To further verify the effects of the composite hydrogel on major organs in vivo, the pathological changes in the lung, liver, spleen, kidney and heart of rats were examined by H&E staining (Figure 5e). The results verify that there was no histotoxicity of the hydrogel in the rats, and the tissues and cell structures of the examined organs (lung, liver, spleen, kidney, and heart) were normal, which provided reliable evidence for the safety of the hydrogel in rats.

These results indicate that the composite hydrogels, in addition to their excellent cytocompatibility and hemocompatibility, also exhibit minimal adverse immune responses and excellent stabilizing power when used as intracorporeal grafts. When soft tissues in organisms are damaged by disease and need to be replaced with tissue engineering materials, TPU‐reinforced PVA hydrogels with outstanding mechanical properties and swelling resistance are competent to replace the biological tissues while maintaining their structure integrity and stability under long‐term loading conditions.

## 3 Conclusion

In summary, TPU‐reinforced PVA hydrogels were successfully prepared through a synergistic approach involving phase separation, wet‐annealing, and quenching. TPU was phase‐separated from the solution and uniformly distributed in the final composite hydrogels. Wet‐annealing and quenching significantly increased the crystallinity of PVA macromolecules and enhanced interfacial adhesion and hydrogen bonding between TPU and PVA. The mechanical properties of PVA_15_‐TPU_8_‐AQ are remarkable, with a tensile strength of 11.19 ± 0.80 MPa, a fracture strain of 1030 ± 106%, and a toughness of 62.67 ± 10.66 MJ m^−3^. Additionally, the composite hydrogel demonstrated excellent crack propagation resistance, with a high fatigue threshold of 1377.83 ± 62.78 J m^−2^. The dense crystalline domains and hydrophobic TPU phases impart the composite hydrogel with superior swelling resistance, maintaining its mechanical properties even after immersion in various aqueous media for 30 days. Furthermore, the composite hydrogel exhibits hemocompatibility and cytocompatibility, as confirmed by in vivo experiments showing no inflammation in the skin tissues or internal organs of rats. These strong, tough, and biocompatible composite hydrogels, with their anti‐swelling and anti‐fatigue properties, hold significant potential for applications in tissue engineering and biomedicine.

## 4 Experimental Section

### Fabrication of Different Hydrogels

PVA and TPU granules were added to DMSO at mass fractions of x% and y%, respectively. The mixture was heated and stirred at 110 °C for 4 h to form a homogeneous solution, referred to as L‐PVA_x_‐TPU_y_. The solution was then poured into a polytetrafluoroethylene mold, cooled to room temperature, and then immersed in water or glycerol for 48 h to obtain hydrogels via one‐step solvent exchange (PVA_x_‐TPU_y_ or Gly‐PVA_x_‐TPU_y_). The organogel was subjected to wet annealing at 120 °C for 30 min, followed by quenching in a large volume of deionized water for 48 h, resulting in the final hydrogels (PVA_x_‐TPU_y_‐AQ).

## Conflict of Interest

The authors declare no conflict of interest.

## Supporting information



Supporting Information

## Data Availability

The data that support the findings of this study are available from the corresponding author upon reasonable request.
