# Supplementary material for: Thermoplastic Elastomer‐Reinforced Hydrogels with Excellent Mechanical Properties, Swelling Resistance, and Biocompatibility
Source: Adv Sci (Weinh). 2025 Feb 7;12(12):2414339. doi: 10.1002/advs.202414339 (PMC11948048; doi:10.1002/advs.202414339)
Supplement: Supplementary file 1 — Supporting Information [file ADVS-12-2414339-s001.docx]

Supporting Information

**Thermoplastic** **Elastomer****-Reinforced Hydrogels with Excellent Mechanical Properties, Swelling Resistance, and Biocompatibility**

Zhanqi Liu^a, 1^, Hechuan Zhang^a, 1^, Ruigang Zhou^b^, Haiyang Gao^c^, Yongchuan Wu^a^, Yuqing Wang^a^, Haidi Wu^a^, Cheng Guan^a^, Ling Wang^d^, Longcheng Tang^e^, Pingan Song^f^, Huaiguo Xue^a^, Jiefeng Gao^*a^

^a^ *School of Chemistry and Chemical Engineering, Yangzhou University, No 180, Road Siwangting, Yangzhou, Jiangsu, 225002, China.*

*^b^College of Veterinary Medicine, Yangzhou University, Yangzhou, 225009, China.*

*^c^* *Department of Chemistry, College of Liberal Arts and Sciences, University of Florida, Gainesville, FL, 32611, America.*

^d^ *School of Chemistry and Chemical Engineering, Anqing Normal University, Anqing 246011, China.*

^e^ *Key Laboratory of Organosilicon Chemistry and Material Technology of Ministry of Education, College of Material, Chemistry and Chemical Engineering, Hangzhou Normal University, Hangzhou, 311121, China.*

^f^ *Centre for Future Materials, University of Southern Queensland, Springfield, QLD, 4350, Australia.*

^1^ These authors contribute equally to this article and should be considered as co-first authors.

*Corresponding author: E-mail address: jfgao@yzu.edu.cn

**Materials**

Polyvinyl alcohol (PVA1799, Aladdin), polyurethane (TPU, Desmopan,1185A), dimethyl sulfoxide (DMSO, Aladdin), hydrochloric acid (HCl, Sinopharm), glutaraldehyde (50 vol%, Aladdin) and deionized water (homemade) were used in this work.

**Mechanical tests**

Dumbbell-shaped hydrogel specimens with the size of 50 mm in length and 4 mm in width were prepared for the mechanical tests which were conducted at room temperature using a universal tensile machine (Instron Model 3367, USA). Prior to testing, the length and thickness of the samples were measured using a digital vernier caliper and a helical side micrometer. The area working region under the stress-strain curve in the tensile test is referred to as toughness.

**Fatigue testing**

The fatigue resistance of hydrogels was evaluated using the single-notch method. Notches with the size of ~1 mm were introduced to the center of the rectangular hydrogel samples (50 × 10 mm^2^). The test was conducted by using a mechanical stretcher with the load cell of 100 N (FULETEST, China). The initial crack expansion of the hydrogel was recorded using a digital camera (AF4915ZTL, Dino-Lite) during continuous stretching cycles without relaxation. It is crucial to conduct the entire experiment under deionized water bath to prevent hydrogel dehydration. The same tensile ratio (λ) was applied to the unnotched specimen to determine strain energy density W in the N^th^ cycle.

$\text{W}\left( \text{λ}\text{, N} \right)\text{=}\int_{\text{1}}^{\text{λ}} \text{S}\text{dλ}$

where S is the stress and. In turn, the energy release rate (G) can be described as:

$\text{G}\left( \text{λ,N} \right)\text{=2k}\left( \text{λ} \right)\text{×c}\left( \text{N} \right)\text{×W}\left( \text{λ,N} \right)$

where $\text{k}\text{=3/}\sqrt{\text{λ}}$ is obtained empirically from the strain variation, c is the crack length, and W represents the integral area of the N^th^ loaded section. The fatigue threshold is linearly extrapolated and verified for 30,000 cycles at this energy release rate.

**Scanning electron microscopy test**

The brittle fracture was occurred for hydrogel samples in liquid nitrogen, and then the samples were freeze-dried using a freeze-dryer to obtain aerogels. A layer of gold was sputtered onto the fracture surface of the aerogel before observation with a scanning electron microscope (Zeiss Supra55, Germany).

**Measurement of water content**

The hydrogel samples were weighed as m_a_ and then dried at 60°C for 72 hours to remove all water. The dried samples were then weighed as m_b_. The water content of samples can be calculated using the following formula:

$$\text{ϕ}_{\text{water}}\text{=}\frac{\text{m}_{\text{a}}\text{-}\text{m}_{\text{b}}}{\text{m}_{\text{a}}}\text{×100\% }$$

**Measurement of DSC**

10 mL of glutaraldehyde solution (50 vol%) and 1 mL of concentrated hydrochloric acid were dropped into 210 mL of deionized water to form a mixed solution, and then the hydrogel samples were immersed into the above solution for 5 min to make the amorphous domains crosslinked (avoid crystallization of the amorphous domains during the drying process). The crosslinked hydrogels were placed in a large amount of water to remove glutaraldehyde and then dried at 37°C for 48h. The dry PVA hydorgels were used for the differential scanning calorimetry (DSC 8500, Perkin Elmer, USA) test, which was conducted under a nitrogen atmosphere at 30 mL/min with a heating rate of 20°C/min, and the heat flow curves of the aerogel were finally obtained in the range of 40°C-250°C. The presence of a narrow peak in the heat flow curve is the melting point of the PVA crystal domains. Integrating the melting peak area on its curve yields the enthalpy (H_crystalline_), the crystallinity of dry hydrogel ($\text{X}_{\text{dry}}$) sample can be calculated by the following equation

$$\text{X}_{\text{dry}}\text{=}\frac{\text{H}_{\text{crystalline}}}{\text{H}_{\text{crystalline}}^{\text{0}}\text{ }}\text{×100\%}$$

where H^0^_crystalline_=138.6 J/g, which represents the enthalpy of melting of 100 wt.% crystalline PVA. and the crystallinity X_swollen_ of the hydrogels in the swollen state can be calculated by the following equation

$$\text{X}_{\text{swollen}}\text{=}\text{X}_{\text{dry}}\text{×}\left( \text{1-}\text{ϕ}_{\text{water}} \right)\text{ }$$

**XRD test**

Diffraction of hydrogel samples with a diameter of 25 mm were recorded in the range of 5°-60° using a diffractometer (D8 Advance, Bruker, Germany). The average size of crystalline domains (D) of PVA hydrogels was calculated using the following Scherrer's equation:

D = kλ/(βcosθ)

Where k, λ, β, θ are the dimensionless shape factor, the wavelength of X-ray diffraction, the full width at half maximum of the peak, and Bragg angle, respectively.

**SAXS test**

The hydrogels were cut into rectangular samples and tested using SAXS test (NanoSTAR, Bruker AXS, Germany). The long period (L) was obtained using the following bragg equation

$$\text{L=}\frac{\text{2π}}{{\text{ }\text{q}}_{\text{max}}\text{ }}$$

where $\text{q}_{\text{max}}$ is the scattering vector corresponding to the highest peak intensity.

**Rheological measurements**

Cylindrical hydrogel samples with a diameter of 25 mm were used to rheological test, and dynamic frequency, oscillatory strain, and time scans were conducted at 20°C using a DHR rheometer (TA, USA) to obtain the variation curves of energy storage modulus (G') and loss modulus (G'').

**ATR-FTIR characterization**

IR spectra of the hydrogel samples were recorded using an infrared spectrometer (Cary610/670, Varian, USA) to analyze the vibrational peaks of the functional groups.

**AFM characterization**

AFM phase images of hydrogel samples were acquired in non-contact mode using an atomic force microscope SPM-9700HT (Shimadzu, Japan).

**Cytotoxicity and hemolysis tests**

To evaluate the in vitro cytotoxicity of the composite hydrogel, L929 cells were chosen to detect cell viability via a CCK-8 kit. Briefly, 3 × 10^3^ cells/well of L929 were seeded onto 96-well plates and then treated with different concentrations of hydrogel extract (5 mg hydrogel in 1 mL of culture medium). After incubation for 24 h, CCK-8 solution was added, and incubated for 2 h, and the cell absorbance was measured at 450 nm with a microplate reader. Cell viability was determined according to the Equation:

$$\text{Cell viability }\left( \text{\%} \right)\text{ }\text{=}\frac{\text{ absorbance}_{\text{sample}}}{\text{ absorbance}_{\text{control}}\text{ }\text{ }}\text{ ×100}$$

A hemolysis test in vitro was used to evaluate the hemocompatibility of PVA_15_-TPU_8_-AQ. In brief, fresh rat blood was centrifugated at 2000 rpm for 10 min and then washed three times with PBS to obtain the purified red blood cells. Subsequently, the red blood cells were diluted to 5% (v/v) RBC, where PVA_15_-TPU_8_-AQ was placed. Note that PBS and deionized water (DW) were chosen as negative control and positive control, respectively. After incubation at 37 °C for 2 h, the sample mixture was centrifuged at 2000 rpm for 10 min, and the hemolytic performance of different treatments were recorded by a camera. A Multiskan FC microplate reader (Thermo, USA) was used to measure the supernatant optical density (OD) value. The hemolysis rate (%) can be obtained according to the following Equation:

$$\text{Hemolysis rate }\left( \text{\%} \right)\text{ }\text{=}\frac{\text{ A}_{\text{sample}}\text{-}\text{A}_{\text{PBS}}}{\text{ A}_{\text{DW}}\text{-}\text{ A}_{\text{PBS}}\text{ }}\text{ }\text{ × 100 }$$

In vivo safety evaluations: To evaluate the biocompatibility of PVA_15_-TPU_8_-AQ, 6 male Sprague−Dawley rats (300 ± 20 g, 6-8 weeks old) were randomly divided into 2 groups (n = 3), including the control and PVA_15_-TPU_8_-AQ groups. After the SD rats were anesthetized by inhaling 3% isoflurane, the back hair was removed with a razor, and a wound of approximately 2 cm in length was made on the back with a scalpel. PVA_15_-TPU_8_-AQ sample was implanted into the back of SD rats, and the major organs and tissue sections were analyzed by H&E staining and Masson staining in the implanted area 7 days later.

**Supplementary Figures**


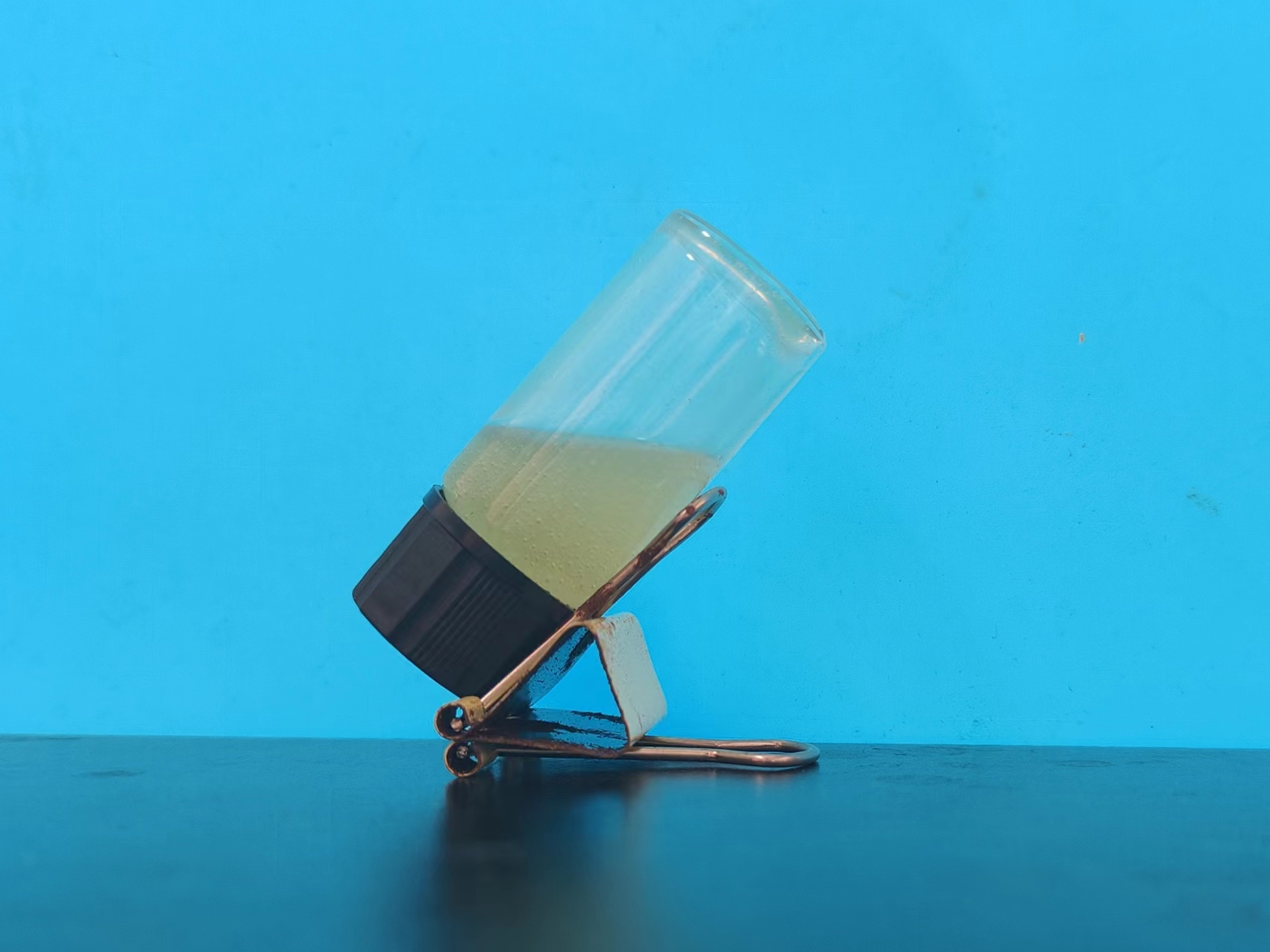


**Figure S1.** Image of homogeneous PVA/TPU DMSO solution.

**Figure S2.** Illustration of microphase separation of polyurethanes.

**Figure S3.** The storage modulus (*G'*) and loss modulus (*G''*) of different hydrogels as a function of oscillation time (ω = 6.28 rad s^-1^, γ = 0.1%, T = 25 ℃).

**Figure S4.** The storage modulus (*G'*) and loss modulus (*G''*) of hydrogels as a function of oscillation strain a) (ω = 6.28 rad s^-1^, T = 25 ℃) and b) angular frequency (γ = 0.1%, T= 25 ℃).

**Figure S5.** SEM image of PVA_15_-TPU_8._

**Figure S6.** SEM image of PVA_15_-TPU_5_-AQ_._

**Figure S7.** Enlarged spectra of the stretching variations of C-O of the hydrogels.

**Figure S8.** Crystal region size of the hydrogels.

**Figure S9.** Crystallinity of hydrogels in dry and swollen states.


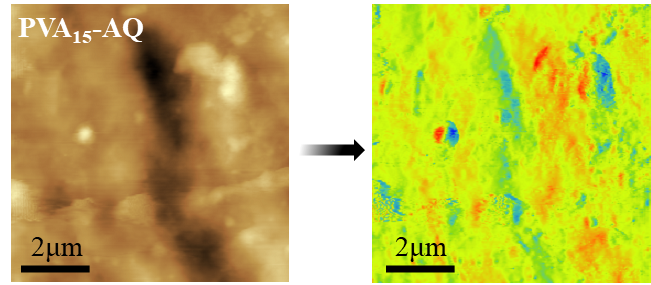


**Figure** **S10.** AFM image and the phase image of PVA_15_-AQ.

**Figure S11.** Stress-strain curves of PVA-TPU and PVA-TPU-AQ hydrogels.


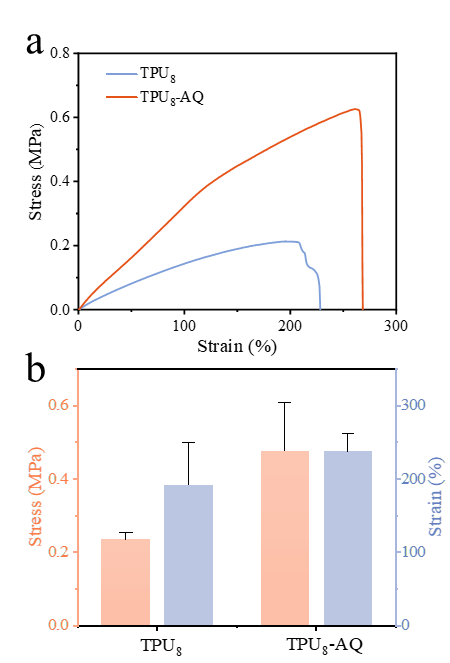


**Figure S12.** Stress-strain curves of TPU_8_ and TPU_8_-AQ.

**Figure S13.** Images of the notch of PVA_15_-TPU_8_-AQ at cycle number 1, 15000 and 30000.

**Figure S14.** a) Stress versus time curve of notched PVA_15_-TPU_8_-AQ with the strain of 70%. b) Stress-strain curves of notched PVA_15_-TPU_8_-AQ in different cycles.


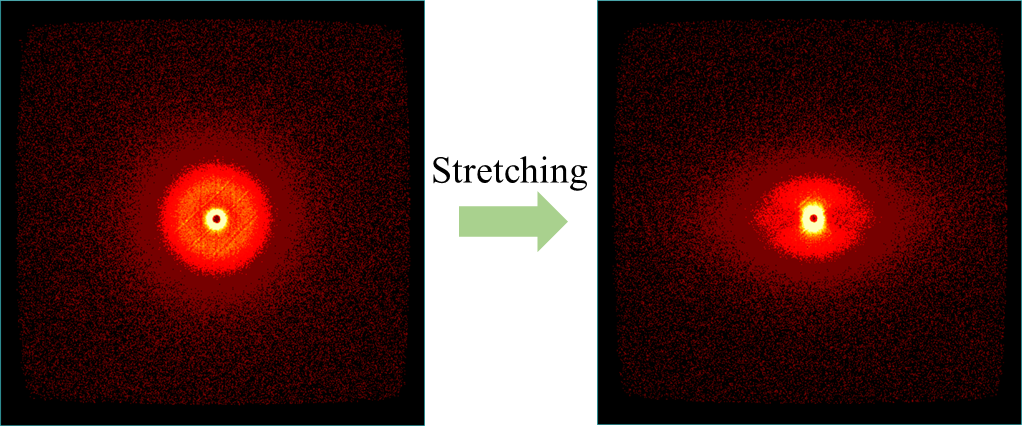


**Figure S15.** SAXS patterns of PVA_15_-TPU_8_-AQ during in-situ stretching to 100%.


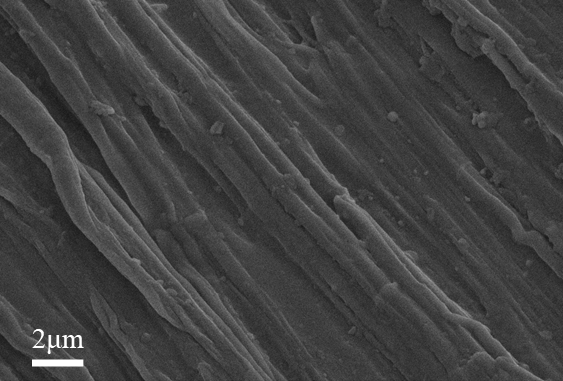


**Figure S16.** SEM image of PVA_15_-TPU_8_-AQ stretched by 100%.


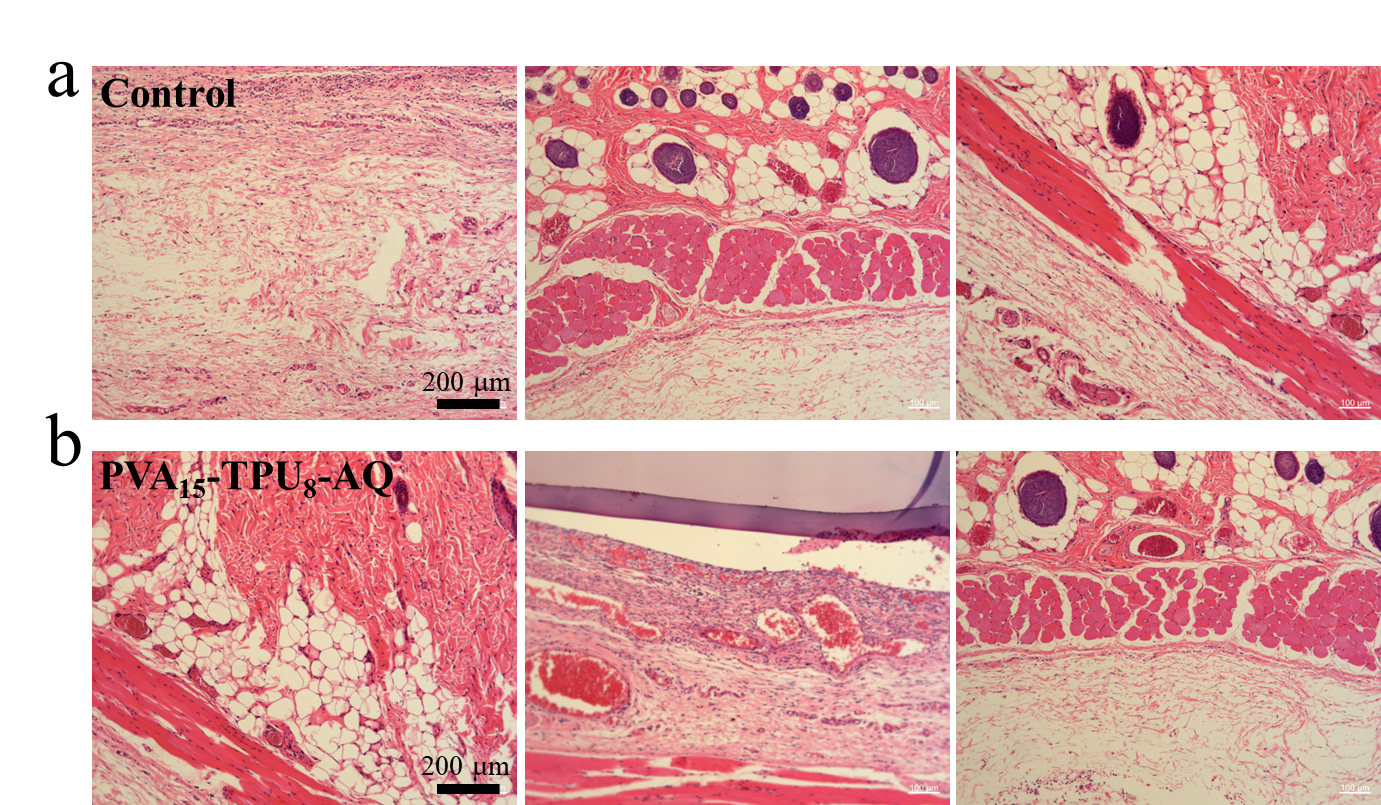


**Figure S17.** H&E of skin tissue in the implantation area for control and PVA_15_-TPU_8_-AQ samples.


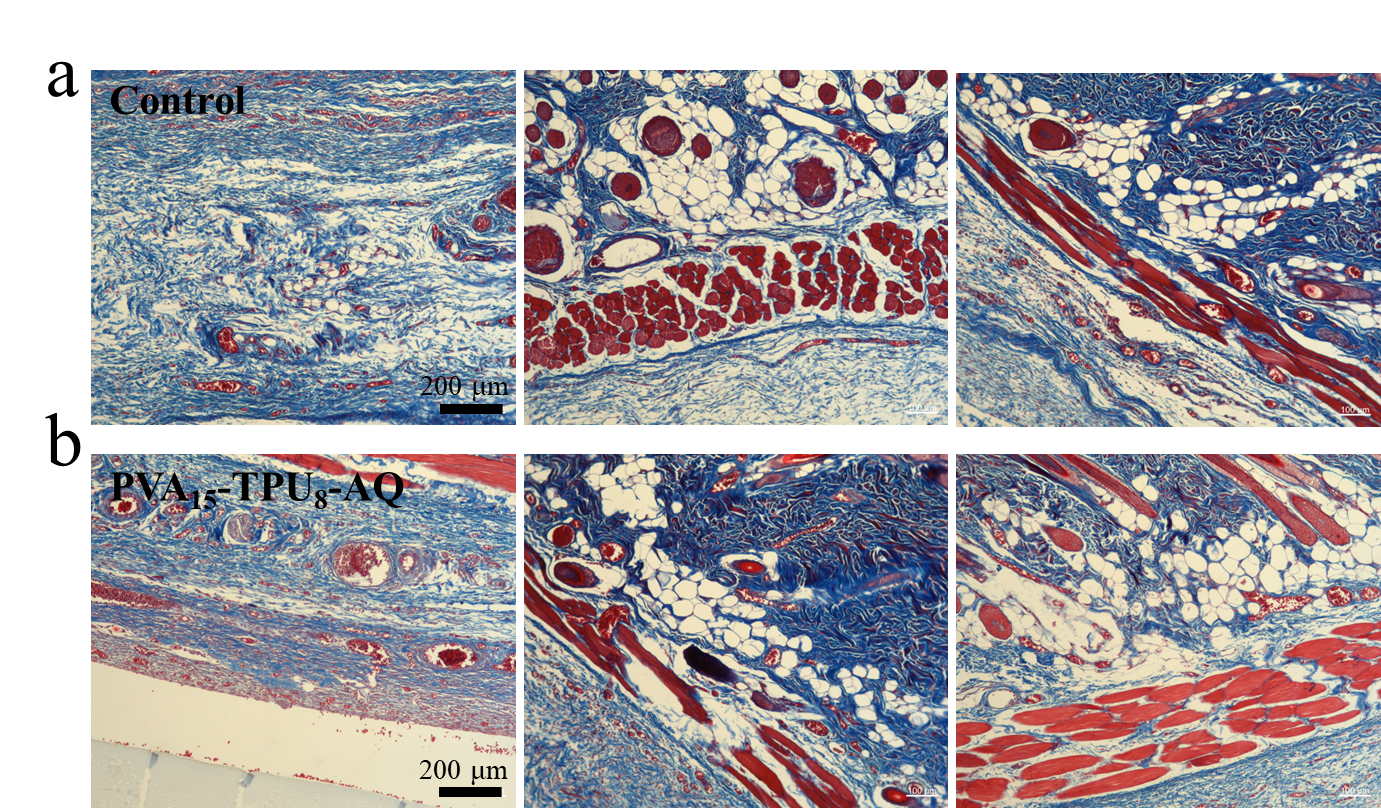


**Figure S18.** Masson of skin tissue in implantation area for control and PVA_15_-TPU_8_-AQ samples.
